# PHDcleav: a SVM based method for predicting human Dicer cleavage sites using sequence and secondary structure of miRNA precursors

**DOI:** 10.1186/1471-2105-14-S14-S9

**Published:** 2013-10-09

**Authors:** Firoz Ahmed, Rakesh Kaundal, Gajendra PS Raghava

**Affiliations:** 1 Bioinformatics Centre, Institute of Microbial Technology, Sector 39-A, Chandigarh, India; 2 National Institute for Microbial Forensics & Food and Agricultural Biosecurity (NIMFFAB), Department of Biochemistry & Molecular Biology, Oklahoma State University, Stillwater, OK 74078, USA; 3 Bioinformatics Laboratory, Plant Biology Division, The Samuel Roberts Noble Foundation, Ardmore, OK 73401, USA

## Abstract

**Background:**

Dicer, an RNase III enzyme, plays a vital role in the processing of pre-miRNAs for generating the miRNAs. The structural and sequence features on pre-miRNA which can facilitate position and efficiency of cleavage are not well known. A precise cleavage by Dicer is crucial because an inaccurate processing can produce miRNA with different seed regions which can alter the repertoire of target genes.

**Results:**

In this study, a novel method has been developed to predict Dicer cleavage sites on pre-miRNAs using Support Vector Machine. We used the dataset of experimentally validated human miRNA hairpins from miRBase, and extracted fourteen nucleotides around Dicer cleavage sites. We developed number of models using various types of features and achieved maximum accuracy of 66% using binary profile of nucleotide sequence taken from 5p arm of hairpin. The prediction performance of Dicer cleavage site improved significantly from 66% to 86% when we integrated secondary structure information. This indicates that secondary structure plays an important role in the selection of cleavage site. All models were trained and tested on 555 experimentally validated cleavage sites and evaluated using 5-fold cross validation technique. In addition, the performance was also evaluated on an independent testing dataset that achieved an accuracy of ~82%.

**Conclusion:**

Based on this study, we developed a webserver PHDcleav (http://www.imtech.res.in/raghava/phdcleav/) to predict Dicer cleavage sites in pre-miRNA. This tool can be used to investigate functional consequences of genetic variations/SNPs in miRNA on Dicer cleavage site, and gene silencing. Moreover, it would also be useful in the discovery of miRNAs in human genome and design of Dicer specific pre-miRNAs for potent gene silencing.

## Background

Dicer is an RNase III enzyme found in almost all eukaryotic organisms and responsible for the processing of pre-miRNAs into miRNAs. In human, Dicer forms a complex with a TAR RNA-binding protein (TRBP) that recognizes and cleaves the pre-miRNA to generate fragments of ~21 base pairs of miRNA:miRNA* duplex [1,2]. Subsequently, the duplex unwinds and miRNA loads into RNA-induced silencing complex (RISC) to execute gene-silencing either through cleavage of the complementary mRNA or by suppression of mRNA translation [3]. Several studies have demonstrated that Dicer is crucial during embryonic development and is involved in number of physiological pathways [4,5]. Human Dicer, a ~200 kDa protein, contains six domains: an ATP/helicase, a DUF283, a PAZ, two RNase III, and a dsRBD domain [6]. All these domains play an important role in the binding and processing of pre-miRNA. Helicase domain is required in the processing of thermodynamically unstable hairpin [7], and the cleavage of double stranded RNA (dsRNA) with blunt or 5'-overhanging termini [8]. The dsRBD domain is involved in the binding of dsRNA. RNase III domains (RIIIa and RIIIb) form heterodimers to make a single active site. RIIIa and RIIIb process the 3p and 5p arms of hairpin respectively and liberate the duplex of miRNA:miRNA* with two nucleotides (nt) 3' overhang [9]. The DUF283 domain adopts a structure similar to dsRBD domain, and possibly plays a role in the target selection [10].

Several biochemical and structural studies have revealed the importance of sequence and structural component in the processing of dsRNA [11,12]. For instance, PAZ domain of Dicer interacts with a 2 nt overhang of 3'-end of dsRNA [13,14]. Length and sequence motifs of overhang influence the Dicer cleavage [13,14]. Genomic variations and mutations in miRNA loci have been reported by several studies [15]. Earlier studies systematically analyzed sequence variations in human pri-miRNAs/pre-miRNA and experimentally discovered that single nucleotide polymorphisms (SNPs) in miR-125a obstruct the processing of pri-miRNA to pre-miRNA [16]. Other studies also showed that SNPs [17] and modification of base A to I in miRNA precursors by adenosine deaminases (ADARs) can impair the processing of miRNA precursors [18]. A recent investigation demonstrated that chemical modification in RNA duplex of >25nt blocks the Dicer processing to generate siRNA [19].

Due to lack of complete knowledge about features of Dicer cleavage sites, investigation of the genetic variations in miRNA and their effect on Dicer cleavage site shift/loss has been impaired. Therefore, it is imperative to study the cleavage site specificity and selectivity of Dicer to gain more insight into RNAi mechanism. In this study, we took the advantage of large dataset available for naturally occurring human specific Dicer substrate pre-miRNA sequences and accordingly, extracted various sequence and structure features associated with the Dicer cleavage site. Finally, these features were implemented in a Support Vector Machine (SVM) framework to develop robust models for predicting the Dicer cleavage site in miRNA hairpin.

## Methods

We retrieved 690 experimentally validated sequences of human miRNAs hairpin from miRBase (version 13) [20]. A Dicer cleavage pattern (positive class) and a non-cleavage pattern (negative class) were extracted from each pre-miRNA sequence. These hairpin sequences were divided into training dataset and independent testing dataset. Training dataset contains 555 sequences of pre-miRNAs and thus having 555 cleavage patterns and 555 non-cleavage patterns. Independent testing dataset contains 135 sequences of pre-miRNAs and constitutes 135 cleavage patterns and 135 non-cleavage patterns.

**Figure 1:**
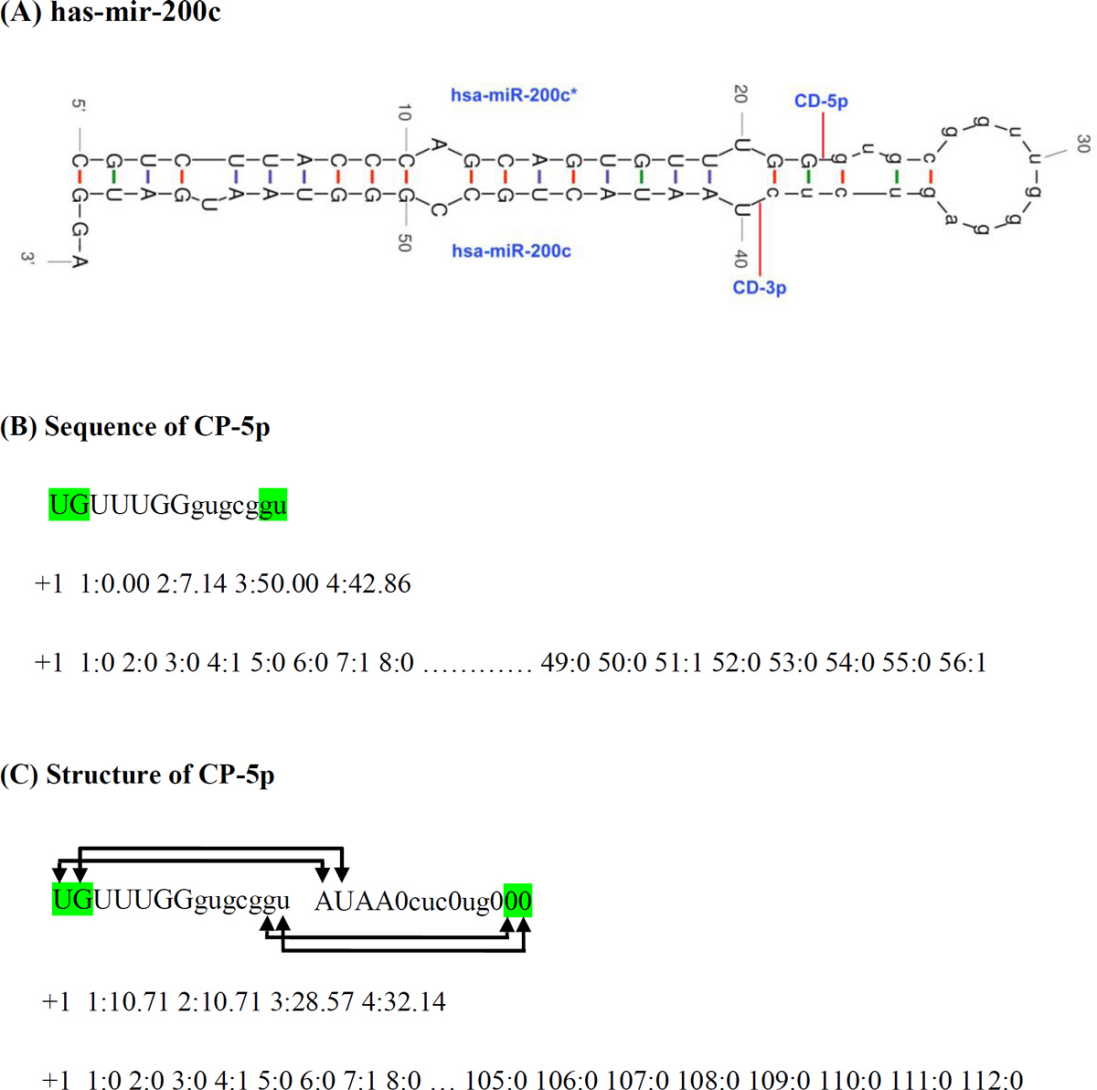
**Schematic diagram of pre-miRNA, hsa-mir-200c, predicted by quikfold software and patterns of Dicer cleavage site at 5p arm**. (A) miR* derived from 5p arm and miR derived from 3p arm of hairpin, bases are represented in capital letter. CD-5p and CD-3p are cleavage sites of Dicer at 5' and 3' arm respectively. (B) Sequence of CP-5p cleavage pattern of 14 nucleotides having cleavage site CD-5p at center. Following each cleavage pattern, features of mononucleotide and binary used as input feature for SVM are given. (C) Structure of CP-5p cleavage pattern of 14 nucleotides having cleavage site CD-5p at center and its partially complementary strand. Base pairs are indicated with arrows. Zero (0) indicates that no base pairing occurs between complementary strands. The pattern of 14+14 is used to generate binary pattern. Mononucleotide having 4, sequence binary pattern having 56, and structure binary pattern having 112 dimensional vector. +1 is the class for cleavage pattern. Binary pattern is represented only for highlighted nucleotides.

**Dicer cleavage and non-cleavage patterns: **Dicer cleavage patterns of 8, 10, 12, and 14 nt length were generated from both 5p and 3p arms of pre-miRNA. The cleavage site present at the center of these patterns and thus termed as positive class. In addition, non-cleavage or negative class of similar length was generated from miRNA after omitting the first six nucleotides adjacent to cleavage site. This is based on the assumption that cleavage site can shift slightly but the chance is rare that Dicer will cut in the middle of mature miRNA. In this study, we used two different sources of input files to generate Dicer cleavage and non-cleavage patterns. (A) We utilized the secondary structure of hairpin given in miRNA.str file downloaded from miRBase. The miRNA.str file contains information of miRNA hairpin base-paired structures, with its Minimum Free Energy (MFE), and position of mature miRNA. The structures were computed using the RNAfold program of the Vienna RNA package [http://www.tbi.univie.ac.at/~ivo/RNA/] (see Figure S1 and S2 in Additional file 1) [21]. (B) We generated the secondary structure of miRNA hairpin by using quikfold server (version 3.0 RNA, http://mfold.rna.albany.edu/?q=DINAMelt/Quickfold), and structure having lowest free energy were taken (Figure 1) [22]. Both tools use dynamic programming to predict RNA secondary structure by free energy minimization using nearest-neighbor energy parameters [23]. More information about Methods is also provided in Additional file 1.

### Features used for development of SVM models

**Nucleotide composition: **The nucleotide composition was counted for the sequences of positive and negative class which is represented by a vector of 4 dimensions for mononucleotide (A, C, G, U); 16 dimensions for dinucleotide (AA, AC, AG, CG, AU.....UU); and 64 dimensions for trinucleotide (AAA, AAC,...., UUU) composition.

**Binary pattern**: In order to have position specific information, we calculated the binary profile of each pattern. In this case, each nucleotide was represented by a vector of 4 dimensions, e.g. A as [1, 0, 0, 0], C as [0, 1, 0, 0], G as [0, 0, 1, 0], and U as [0, 0, 0, 1]. The secondary structure of miRNA precursors frequently contains internal loop/bulge in those regions where one arm has extra inserted bases with no counterparts in the opposite arm. A recent study indicates that the loop/bulge structure also play an important role in the selection of Dicer cleavage site 11 The absence of nucleotides in the sequence and structure taken from miRNA.str file were denoted by "-" (Additional file 1: Figure S1 and S2), while lack of nucleotide in the secondary structure of quikfold was denoted by "0" (Figure 1C). Both "-" and "0" were represented by [0, 0, 0, 0] in the binary pattern, though, it represents a new feature of loop/bulge. Therefore, in order to incorporate new feature of loop/bulge in the structure, we also used an extended binary pattern in which the nucleotides were represented as a vector of 5 dimensions: A as [1, 0, 0, 0, 0]; C as [0, 1, 0, 0, 0]; G as [0, 0, 1, 0, 0]; U as [0, 0, 0, 1, 0]; and the loop/bulge ("0" or "-") as [0, 0, 0, 0, 1].

### Support vector machine and non-redundant 5-fold cross validation

In this study, various SVM models have been developed using the SVM^light ^V5.0 package [24]. SVM is a state-of-the-art machine learning technique that has been extensively applied in the areas of pattern recognition, regression and classification problems in various fields of science and engineering, for example: predicting protein subcellular localization [25-27], protein secondary structure prediction [28], disease forecasting [29], structure prediction [28,30], antibacterial peptides [31,32], and polyadenylation signal prediction in mRNA [33]. In the present study, we optimized the SVM parameters to achieve the best performing models on training dataset using a non-redundant 5-fold cross validation approach.

In non-redundant 5-fold, homologous sequences were kept in one set according to the miFam.dat file of miRBase to prevent positive bias [34]. It is known that sequences of miRNA hairpin show similarity with each other. Therefore, similar sequences of miRNA were categorized together into one family. The information about miRNAs and their family are given in miFam.dat file. For SVM optimization, we have used RBF kernel in combination with different parameters; g ∈[0.001, 0.01, 0.1], c ∈ [1,2,3,....,10] and j ∈ [1,2,3,....,10]. SPSS SmartViewer version 11 on Windows machine has been used for ROC (Receiver Operating Characteristic) analysis.

### Performance measures

The performance of prediction models was measured using: i) sensitivity (Sn), ii) specificity (Sp), iii) accuracy (Ac), and iv) Matthews correlation coefficient (Mc). Following equations were used to calculate these parameters:

$$Sensitivity_{}=\frac{TP}{TP+FN}\times 100$$

$$Accuracy_{}=\frac{TP+TN}{TP+FP+TN+FN}\times 100$$

$$Specificity_{}=\frac{TN}{TN+FP}\times 100$$

$$MCC=\frac{\left(TP\right)\left(TN\right)-\left(FP\right)\left(FN\right)}{\sqrt{\left[TP+FP\right]\left[TP+FN\right]\left[TN+FP\right]\left[TN+FN\right]}}$$

Where TP, FN, TN, and FP are refers to true positive, false negative, true negative and false positive, respectively.

## Results

In this study, the 14 nt Dicer cleavage patterns taken from 5p arm are referred to as CP-5p and cleavage site present at the middle of this pattern is referred to as CD-5p (Figure 1). Similar convention of cleavage patterns is also used for 3p arm and referred to as CP-3p, and CD-3p (see Figure S1 and S2 in Additional file 1). The composition and binary pattern features were generated for the cleavage and non-cleavage patterns and used as input for developing the SVM models. For sequence-based models, we have taken the feature associated with cleavage pattern, while in structure-based model information about cleavage pattern, its base pairs as well as bulges and loop are also considered. As generally, there is a 2 nt overhang at the 3'-end of miR:miR* duplex; using this information, the cleavage sites at 3p arm can be found from the 5p arm and vice versa.

## Prediction using features calculated by RNAfold (miRNA.str file)

**Sequence-based models: **At first, we extracted the pattern of Dicer cleavage sites from 5p arm and achieved the highest accuracy of 62.61, 62.88, 59.73 and 74.50% for mono-, di- and tri-nucleotide composition and binary pattern, respectively (Table 1). While for 3p arm, we achieved a highest accuracy of 65.14, 63.15, 62.79 and 67.84% for mono-, di-, tri-nucleotide composition and binary pattern, respectively (Table 1).

**Table 1:** Performance of 'RNAfold' derived sequence-based SVM models for Dicer cleavage sites at 5p and 3p arm.

Features	Window size	CD-5p (sequence-based)				CD-3p (sequence-based)			
		Sn	Sp	Ac	Mc	Sn	Sp	Ac	Mc
**Mono**	8	58.20	61.80	60.00	0.20	58.38	59.28	58.83	0.18
	10	63.78	58.92	61.35	0.23	60.36	61.44	60.90	0.22
	12	61.08	63.96	62.52	0.25	64.68	59.46	62.07	0.24
	**14**	**60.36**	**64.86**	**62.61**	**0.25**	**63.78**	**66.49**	**65.14**	**0.30**

**Dinuc**	8	59.82	59.82	59.82	0.20	61.80	52.25	57.03	0.14
	10	60.54	58.20	59.37	0.19	58.74	52.97	55.86	0.12
	12	61.44	56.40	58.92	0.18	60.90	61.62	61.26	0.23
	**14**	**60.72**	**65.05**	**62.88**	**0.26**	**60.36**	**65.95**	**63.15**	**0.26**

**Trinuc**	8	60.90	57.48	59.19	0.18	58.92	56.04	57.48	0.15
	10	55.14	61.62	58.38	0.17	59.82	53.15	56.49	0.13
	12	58.92	55.14	57.03	0.14	63.24	57.12	60.18	0.20
	**14**	**60.72**	**58.74**	**59.73**	**0.19**	**64.68**	**60.90**	**62.79**	**0.26**

**Binary**	8	60.54	61.62	61.08	0.22	65.23	65.05	65.14	0.30
	10	61.26	63.78	62.52	0.25	67.75	63.06	65.41	0.31
	**12**	67.57	65.05	66.31	0.33	**69.55**	**66.13**	**67.84**	**0.36**
	**14**	**71.89**	**77.12**	**74.50**	**0.49**	70.99	63.96	67.48	0.35

Models were developed using composition and binary pattern of different window sizes. Patterns were taken from sequence of Dicer cleavage site of 5p arm (sequence of CP-5p) and 3p arm (sequence of CP-3p) using miRNA.str data.

CD-5p: Dicer cleavage site at 5p arm, CD-3p: Dicer cleavage site at 3p arm, Mono: Mononucleotide, Dinuc: Dinucleotide, Trinuc: Trinucleotide, Binary: Binary pattern, Sn: sensitivity, Sp: specificity, Ac: accuracy, Mc: Matthews correlation coefficient.

**Structure-based models: **In addition to the sequence of Dicer cleavage pattern, we also considered the information of its complementary strand. Using Dicer cleavage site for 5p arm, we achieved the highest accuracies of 71.80, 66.76, 65.14 and 77.03% for mono-, di- and tri-nucleotide composition and binary pattern, respectively (Table 2). Whereas, the Dicer cleavage site taken from 3p arm achieved a best accuracy of 64.50, 63.24, 60.72, and 70.36% for mono-, di- and tri-nucleotide composition and binary pattern, respectively (Table 2).

**Table 2:** Performance of 'RNAfold' derived structure-based SVM models for Dicer cleavage sites at 5p and 3p arm.

Features	Window size	CD-5p (structure-based)				CD-3p (structure-based)			
		Sn	Sp	Ac	Mc	Sn	Sp	Ac	Mc
**Mono**	8	62.88	65.59	64.23	0.28	62.88	57.30	60.09	0.20
	10	67.21	66.85	67.03	0.34	63.24	63.06	63.15	0.26
	12	71.53	69.01	70.27	0.41	59.28	67.21	63.24	0.27
	**14**	**68.83**	**74.77**	**71.80**	**0.44**	**61.62**	**67.39**	**64.50**	**0.29**

**Dinuc**	8	61.98	63.24	62.61	0.25	57.84	59.46	58.65	0.17
	10	63.60	63.78	63.69	0.27	59.64	58.92	59.28	0.19
	12	69.19	62.34	65.77	0.32	63.96	62.52	63.24	0.26
	**14**	**68.83**	**64.68**	**66.76**	**0.34**	**65.41**	**61.08**	**63.24**	**0.27**

**Trinuc**	8	54.77	63.42	59.10	0.18	57.84	55.32	56.58	0.13
	10	63.24	59.82	61.53	0.23	57.84	60.00	58.92	0.18
	12	63.24	63.60	63.42	0.27	60.54	57.12	58.83	0.18
	**14**	**67.39**	**62.88**	**65.14**	**0.30**	**56.76**	**64.68**	**60.72**	**0.22**

**Binary**	8	65.23	65.41	65.32	0.31	65.23	70.45	67.84	0.36
	10	67.57	67.57	67.57	0.35	72.07	62.88	67.48	0.35
	12	72.79	69.91	71.35	0.43	71.89	68.11	70.00	0.40
	**14**	**77.84**	**76.22**	**77.03**	**0.54**	**70.99**	**69.73**	**70.36**	**0.41**

Models were developed using composition and binary pattern of different window sizes. Patterns were taken from structure of Dicer cleavage site of 5p arm (structure of CP-5p) and 3p arm (structure of CP-3p) using miRNA.str data.

## Prediction using features calculated by quikfold

**Sequence-based models: **For prediction using the miRNA hairpin by quikfold server, the cleavage site at 5p arm was considered only because of its better discrimination feature compared to the 3p arm (Table 1 and 2). Here, we achieved an accuracy of 58.92, 59.55, 60.72, and 66.13% for mono-, di- and tri-nucleotide composition, and binary pattern, respectively for sequence-based features (Table 3).

**Table 3:** Performance of 'quikfold' derived sequence- and structure-based SVM models for Dicer cleavage sites at 5p arm.

Features	Window size	CD-5p (sequence-based)				CD-5p (structure-based)			
		Sn	Sp	Ac	Mc	Sn	Sp	Ac	Mc
**Mono**	8	56.76	56.94	56.85	0.14	69.37	70.45	69.91	0.40
	10	59.10	56.40	57.75	0.16	75.68	77.48	76.58	0.53
	12	54.05	57.84	55.95	0.12	77.84	83.06	80.45	0.61
	14	**58.20**	**59.64**	**58.92**	**0.18**	**81.44**	**82.70**	**82.07**	**0.64**

**Dinuc**	8	58.92	59.46	59.19	0.18	65.95	67.03	66.49	0.33
	10	55.50	61.08	58.29	0.17	67.39	67.57	67.48	0.35
	12	59.82	56.76	58.29	0.17	69.73	74.41	72.07	0.44
	14	**64.14**	**54.95**	**59.55**	**0.19**	**73.33**	**73.33**	**73.33**	**0.47**

**Trinuc**	8	61.98	52.43	57.21	0.14	60.36	59.10	59.73	0.19
	10	55.32	50.45	52.88	0.06	66.31	62.34	64.32	0.29
	12	57.48	56.58	57.03	0.14	65.77	65.41	65.59	0.31
	14	**61.26**	**60.18**	**60.72**	**0.21**	**68.65**	**65.41**	**67.03**	**0.34**

**Binary**	8	62.70	58.92	60.81	0.22	69.55	78.02	73.78	0.48
	10	61.26	59.46	60.36	0.21	76.40	80.36	78.38	0.57
	12	65.41	63.42	64.41	0.29	82.16	81.26	81.71	0.63
	14	**62.52**	**69.73**	**66.13**	**0.32**	**85.23**	**87.21**	**86.22**	**0.72**

Models were developed using composition and binary pattern of different window sizes. Patterns were taken from sequence of Dicer cleavage site of 5p arm (sequence of CP-5p) and structure of Dicer cleavage site of 5p arm (structure of CP-5p).

**Figure 2:**
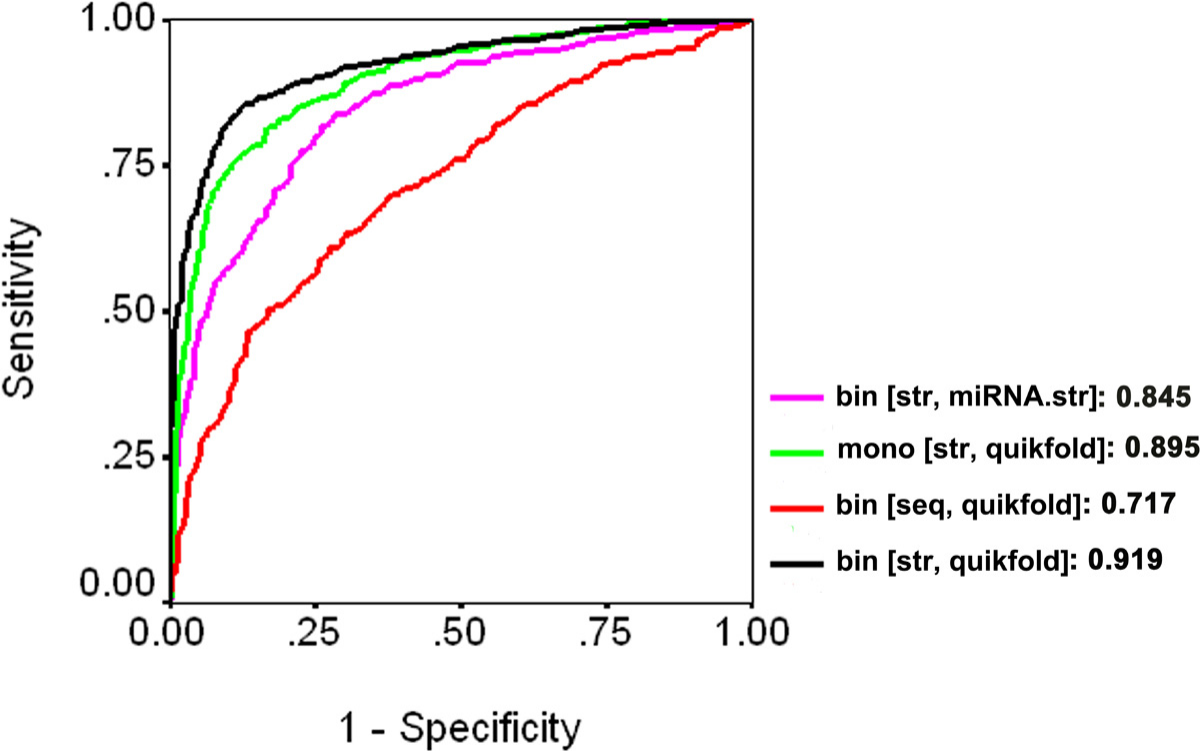
**Performance of various SVM models for Dicer cleavage site at 5p arm (CD-5p) shown by ROC plots**. Bin [str, miRNA.str]: binary feature used for structure of CP-5p taken from miRNA.srt. Mono [str, quikfold]: mononucleotide composition used for structure of CP-5p taken from quikfold. Bin [seq, quikfold]: binary feature used for sequence of CP-5p taken from quikfold. Bin [str, quikfold]: binary feature used for structure of CP-5p taken from quikfold. The value indicates the AUC for the corresponding model.

**Structure-based models: **The structure-based Dicer cleavage site for 5p arm achieved a highest accuracy of 82.07, 73.33, 67.03, and 86.22% for mono-, di- and tri-nucleotide composition, and binary pattern, respectively (Table 3 and Table S1 in Additional file 1). The performance of different models was also tested by ROC curve, which is plotted as a graph of true positive rate (sensitivity) against a function of false positive rate (1-specificity). Figure 2 shows that AUC (area under curve) is the highest (0.919) for structure-based feature (generated from quikfold) using binary pattern.

**Performance on independent testing dataset: **We also evaluated the performance of our two best models (mononucleotide, and binary pattern of quikfold structure-based pattern) on an independent testing dataset. The independent testing dataset contains 135 sequences of miRNA hairpin. All sequences in the independent testing dataset are taken from the miRNA family not included in the training dataset. This is to ensure that the sequence similarity between miRNA hairpins from training and independent testing data set is kept to the minimal, which is essential to know the correct and unbiased performance of a model. On independent testing dataset, we achieved an accuracy of 78.88 and 78.15%, and AUC of 0.861 and 0.872 for mononucleotide and binary pattern, respectively (Additional file 1: Figure S3 and Table S2).

**Performance using hybrid and extended binary features: **In an extended approach, we also employed features of quikfold structure to further increase the accuracy. Initially, various hybrid models were developed using a combination of two or more features at a time (*viz*. mono-, di- and tri-nucleotide compositions and binary pattern) but the accuracy was not improved (data not shown). Finally, a best model (***Model 1***) was designed that achieved a maximum accuracy of 86.40% at 84.32% sensitivity and 88.47% specificity with AUC value of 0.922 by using extended binary feature (Table 4 and Table S3 in Additional file 1). The model was also tested on an independent testing dataset that achieved an accuracy of 81.85% with AUC of 0.889 (Additional file 1: Table S4).

**Table 4:** Performance of different WEKA methods for Dicer cleavage prediction and their comparison with SVM model.

Methods	Sn	Sp	Ac	Mc	AUC
**SVM^light ^(*Model 1*)**	**84.32**	**88.46**	**86.40**	**0.730**	**0.922**
Random Forest	80.54	90.81	85.68	0.717	0.921
Naïve Bayes	81.26	86.30	83.78	0.676	0.909
Simple CART	81.08	85.40	83.24	0.665	0.879
REP Tree	80.54	81.08	80.81	0.616	0.872
Random Tree	69.54	74.05	71.80	0.436	0.752

All models were used for the prediction of cleavage site at 5p arm by using 'extended binary pattern' feature of 14 nt window taken from 'quikfold' derived structure (structure of CP-5p). Models were developed on training dataset using non-redundant 5-fold cross validation techniques.

Sn: sensitivity, Sp: specificity, Ac: accuracy, Mc: Matthews correlation coefficient. AUC: area under curve.

**Development of models using Weka package: **WEKA is a powerful toolkit for data mining and use for classification of biological data [35,36]. In this study, we also checked the performance of models developed based on the classifiers implemented in Weka version 3.6.9 (other than SVM). All models were evaluated using the same non-redundant 5-fold cross validation technique, data and features as used for developing the SVM ***Model 1***. It was found that though the performance of Random Forests is better than other algorithms of Weka, its accuracy (85.68%) is slightly lower than the accuracy (86.40%) of SVM^light ^(Table 4).

**Performance of extended binary feature on overlapping patterns (*Model 2*): **In the previous models, we used 555 pre-miRNA sequences to generate 555 positive and 555 negative patterns for developing a SVM classifier. This means that from a hairpin, we extracted one cleavage site and one non-cleavage site. However, in reality there could be one (or few) cleavage site(s) and the rest sites be considered as non-cleavage in a hairpin [37-39]. Thus, we extracted the overlapping patterns of 14 base pairs along the hairpin-stem region. The patterns, which contain the cleavage site in the middle (between 7^th ^and 8^th ^base pair) were considered as positive and the rest as negative patterns. In this way, the whole data generated a total of 555 positive patterns and 18662 negative patterns with a ratio of 1:33. Then extended binary pattern was calculated for the new datasets and the same non-redundant 5-fold cross validation was used to develop the SVM model (***Model 2***). Performance of ***Model 2 ***using this new dataset showed that at a sensitivity of 62.34%, the specificity, accuracy and MCC are 88.39%, 87.64% and 0.25, respectively (Additional file 1: Table S5). This performance of ***Model 2 ***with low sensitivity and high specificity values might be due to the use of imbalanced datasets where negative instances outnumber the positive instances. Therefore, to check the effect of imbalanced dataset, we randomly selected 555 negative patterns out of 18662 and used with the 555 positive patterns to develop another SVM model, ***Model 2^balanced^***. We observed a better performance of ***Model 2^balanced ^***(sensitivity: 79.46, specificity: 73.15, accuracy: 76.31, MCC: 0.53) compared to the performance of ***Model 2 ***(Additional file 1: Table S6).

**Comparison between performances of *Model 1 *and *Model 2 *on exclusive miRBase 14 dataset: **Above study was performed using the datasets from miRBase 13. To evaluate the performance of our models, we have taken new entries of pre-miRNA from miRBase version 14 and named them as exclusive miRBase 14 data. We retrieved 32 human pre-miRNA entries, but two of them (hsa-mir-212 and hsa-mir-220c) form multiple loops in their predicted secondary structure, thus, we took only 30 human pre-miRNAs for assessment (Additional file 1: Table S7). We predicted the Dicer cleavage sites of these pre-miRNAs and selected the top three cleavage sites based upon highest score, and compared them with the actual cleavage site using a Position Shift Error (PSE). PSE is calculated by abstracting the actual position from the predicted one, which indicates the extent of deviation in the cleavage site prediction. For instance, PSE 0 indicates that the predicted site is similar to actual, -1 means the predicted site is one nt upstream from the actual and 1 indicates that the predicted site is one nt downstream from the actual. Average PSE is calculated for each of the 1^st^, 2^nd ^and 3^rd ^top score sites in which we add the absolute value (-1 as 1; -2 as 2) of PSE and divide by the total number of sequences. The performance of ***Model 1 ***showed that the average PSE of 1^st ^top score is 3.1 including PSE 0 (8), PSE 1 (6), PSE 2 (5), and PSE 3 (2) (Additional file 1: Table S8). The cleavage site of hsa-mir-548q is very different from the real, thus, excluding this average PSE becomes 2.52. However, performance of ***Model 2 ***showed an average PSE of 3.03 on the 1^st ^top score including PSE 0 (6), PSE 1 (7), PSE 2 (6), and PSE 3 (1). By excluding the hsa-mir-548q, the average PSE becomes 2.48 (Additional file 1: Table S9). Results of ***Model 1 ***also demonstrates that it can predict the accurate cleavage sites in 17 pre-miRNAs, while ***Model 2 ***predicts only 14 accurate sites out of the 30 sequences when considering top three predicted sites (Additional file 1: Table S8, S9). Overall, the results show that the performance of SVM ***Model 1 ***is better and robust. Therefore, ***Model 1 ***has been implemented in the development of PHDcleav server. A description of PHDcleav server including *input sequence*, *threshold parameter*, and *output results *are provided in Additional file 1.

## Application of PHDcleav

Several studies have identified single nucleotide polymorphisms (SNPs) in the miRNAs and their target genes, which possibly affect miRNA biogenesis, expression of target genes, and contribute to diseases [15,40-42]. Jazdzewski *et al *found that polymorphism in hsa-mir-146a reduces the formation of pre- and mature-miRNA [40]. PHDcleav could be used in genome-wide investigations of miRNA-related polymorphisms and their consequence on Dicer processing site. In this study we have analyzed the effect of SNPs on Dicer cleavage site using miRNA hairpins taken from miRvar, a database of SNPs within miRNA [43]. We observed that SNPs in hairpin could potentially affect the Dicer cleavage site by various ways. SNPs in hsa-mir-335 and hsa-mir-941-3 results in loss and alteration in cleavage site, respectively (Table 5). While SNPs in hsa-mir-196a-2, hsa-mir-570, and has-mir-650 did not affect their cleavage site (Table 5). Differences in the effect on cleavage site may be due to location of SNPs in hairpin and the base composition that can ultimately change the structural properties of pre-miRNA. A study also utilized PHDcleav and reported similar observation [43]. Shift in the Dicer cleavage site would change the terminal sequences and thermodynamic stability of duplex end, which may result in the loading of miRNA* strand into RISC and cause miRNA* associated off-target silencing. Therefore, a previous study used RISCbinder tool [34] and found variation in Dicer cleavage site may change the loading strand into RISC [43]. Alteration in Dicer cleavage sites can also change the seed region (2-8 nt from 5'-end) of mature miRNA which plays a critical role in the target recognition and consequently may change the repertoire of miRNA targets [44].

**Table 5:** Analysis of functional consequences of SNPs on Dicer processing sites using PHDcleav.

miRBase 19 ID	Reference		Genetic variant					
		
	Cleavage Position	PHDcleav Score	Variation	dbSNP ID	SNP location on hairpin	Cleavage Position	PHDcleav Score	Effect on Dicer cleavage site
hsa-mir-196a-2	47	2.326	g.78C>T	rs11614913	Mature	47	2.326	remain same
	**46**	**2.055**				**46**	**2.055**	
	48	1.614				48	1.614	

hsa-mir-335	39	2.855	g.39T>C	MIR335_00001*	Mature	37	1.653	loss of site
	**38**	**1.367**				36	1.337	
	37	1.338				35	1.027	

hsa-mir-570	**46**	**2.025**	g.34T>C	rs9860655	Stem	**46**	**2.025**	remain same
	45	1.126				45	1.126	
	39	0.908				40	0.994	

hsa-mir-650	35	1.366	g.71C>G	rs59996397	Stem	35	1.366	remain same
	**36**	**1.349**				**36**	**1.349**	
	38	1.271				38	1.271	

hsa-mir-941-3	57	2.095	g.69C>G	rs12625445	Mature	57	2.398	altered
	**55**	**1.011**				***56***	***1.034***	
	56	0.750				55	1.023	

Top three scores of PHDcleav with their corresponding cleavage sites are given in the table, higher score indicates most probable Dicer cleave site. miRBase annotated canonical cleavage site is shown in bold, while altered cleavage site is shown in bold and italics.

*miRvar DB-ID [43].

Sequence specific gene silencing can be induced by expressing small hairpin RNA (shRNA) or artificial miRNA (amiRNA) into the cells or tissues [45]. The amiRNA is designed by exploiting the backbone of a miRNA hairpin by replacing miRNA/miRNA* with siRNA duplex [46]. PHDcleav tool can also be used to optimize and design more accurate site for Dicer cleavage in the shRNA/amiRNA. The Dicer cleaves at position 28 nt on 5'-arm and 46 nt on 3'-arm of hsa-mir-199a-1. PHDcleav predicted the same positions for Dicer cleavage with a highest score of 1.072. Therefore, PHDcleav can also be used to optimize and design a better amiRNA with a predefined Dicer cleavage site using hsa-mir-199a-1 backbone. Human population also contains disease isoform/SNPs genes and a tool, desiRm, has been developed for silencing such genes by mismatch siRNA [47]. In addition, the role of siRNAs against HIV infection has also been well documented in HIVsirDB [48]. Therefore, PHDcleav could also be used with other resources such as desiRm and HIVsirDB to design shRNA/amiRNA to target multiple sequences in order to prevent viral escape [49]. Furthermore, PHDcleav could also be combined with other tools to predict highly accurate miRNA in the RNA-seq data as shown in one of the recent studies [50].

## Discussion

RNAi pathway involves step-by-step processing of RNA hairpin and finally releases a functional miRNA for target silencing. In order to develop robust algorithms for any pathway, it is imperative to develop a method for each step involved in that pathway such as prediction of peptides binding to MHC class [51,52]. Before 2007, computational studies were mainly focused on predicting miRNA and its target [53]. Afterwards, a method was developed to predict Drosha cleavage site wherein its relevance in improving the prediction of miRNA has been shown [54]. However, a big population of miRNAs are also generating from intronic regions without the involvement of Drosha [55-57]. Unlike Drosha, almost all miRNA hairpins and dsRNAs are processed by Dicer to generate the mature miRNA and siRNA, respectively. However, computational method for the prediction of Dicer cleavage site is still not available, which could be useful to know the cleavage site in a potential miRNA hairpin sequence.

In this study, we have developed an accurate model for predicting the Dicer cleavage sites in miRNA precursors. Initially, we developed SVM models using the sequence information taken from miRNA.str and achieved an accuracy of 65.14% for mononucleotide and 74.50% for binary pattern (Table 1). The performance of dinucleotide-based classifier is nearly equal or less than a mononucleotide-based classifier, while for trinucleotide is slightly lower than the both. This decrease in accuracy could be attributed to the fact that there is no specific long motif associated with a cleavage site, thus, an increase in the content of information in di- and tri-nucleotide doesn't improve the discriminatory features between cleavage and non-cleavage sites. The performance of binary-based method is better than composition-based method because it contains the position specific nucleotide information. In addition, we found that the performance of 14 nt window size is better than 8, 10, and 12 nt because the content of nucleotide information around the cleavage site is more.

Furthermore, we implemented secondary structure information of Dicer cleavage sites, which captures base pairing information and topological restrain of cleavage site to develop models. In our previous study, we have also shown that incorporating structural information of RNA could increase the prediction accuracy of guide strand [34]. Since the enzymes of all the members of dsRNA specific RNase III family recognize the structure of substrate [16,17,19,58-60], we used RNA secondary information around the cleavage sites taken from miRNA.str. Incorporation of this information led to 71.80% accuracy for mononucleotide and 77.03% for binary pattern (Table 2). It is important to note that we achieved similar accuracy for CD-3p from both the sequence and structure-based models (Table 1 and 2). Moreover the accuracy achieved by CD-5p for sequence-based to structure-based has significantly improved *i.e*. 62.61% to 71.80% for mononucleotide and 74.50% to 77.03% for binary pattern. Thus, we infer from this study that Dicer has better discriminatory feature at CD-5p over CD-3p and it would be interesting to explore those structural determinants experimentally. Very recently, it has been demonstrated the importance of 5'-end of pre-miRNA and its interaction with Dicer for efficient processing [61].

Since the discrimination features at 5p arm are better, we developed additional models using pre-miRNAs predicted by another software quikfold. Models developed using the sequence pattern achieved highest accuracy of 66.13% while the structural pattern achieved an accuracy of 86.22% for binary pattern method. Comparing the accuracies achieved by RNAfold (miR.str) and quikfold, we observed that performance of sequence-based model taken from miR.str is better than quikfold model, while the structure-based model is better for quikfold. The variation between the two models could be due to the difference in the input information taken from these two methods. The sequence pattern in miR.str, if present in complementary strand, also contains information of bulges, while sequence pattern of quikfold contains only nucleotide information. The secondary structures taken from quikfold have information of around all 14 nt cleavage site, its base pairing nucleotide in complementary region as well as bulges and loops (Figure 1, and Figure S1 in Additional file 1). The model developed using extended binary pattern feature has achieved better prediction accuracy, and the performance is also better on an independent testing dataset (Additional file 1: Table S3 and S4). This slight gain in the performance of extended binary pattern over the normal binary pattern is due to incorporating an additional feature of loop/bulge of hairpin structure in ***Model 1***. The information of loop/bulge was encoded as "00001" in extended binary pattern which was lacking in binary pattern. This result is also supporting a recent finding which indicates the role of loop/bulge structure in the selection of Dicer cleavage site [11]. Moreover, the models developed using other methods of Weka are indicating that though the performance of Random Forest is satisfactory, the SVM is a better classifier for predicting the Dicer cleavage site (Table 4). Some other studies have also reported SVM as the best classifier for prediction [36].

Additionally, we also developed ***Model 2 ***by considering one positive pattern and the rest as negative patterns from each of the pre-miRNAs and achieved an accuracy of 87.64% (Additional file 1: Table S5). Performance of ***Model 2 ***is lower than that of ***Model 1*; **this might be due to two reasons: (1) use of imbalanced ratio of positive and negative datasets in ***Model 2 ***(ratio 1:33). Studies have shown that prediction performance drops when SVM models are developed on highly skewed training dataset [62-64]. Moreover, ***Model 2^balanced ^***developed on a balanced dataset of positive and negative patterns achieved a better performance than ***Model 2 ***(Additional file 1: Table S5, S6); (2) Most importantly, a slight variation at the site of cleavage by Dicer has been reported by high-throughput sequencing, that generates various isomers of miRNA [37-39,44]. Thus in overlapping patterns of our data, there is a slight variation in the cleaving and non-cleaving patterns with a difference of 1-3 bp, which makes it difficult to discriminate between positive and negative patterns. However, prediction threshold can be increased to get higher specificity (higher confidence) values, but with a compromise on sensitivity. Furthermore, the assessment of ***Model 1 ***and ***Model 2 ***on 30 exclusive pre-miRNA datasets has been shown in PSE which indicates that the performance of ***Model 1 ***is robust and better, and thus implemented into the PHDcleav server (Additional file 1: Table S8 and S9). Under the '*applications*' section, we have discussed few examples and area of work where the PHDcleav tool could be applied successfully.

In our study, we have included all the experimental data available in miRBase. Therefore, we hypothesize that our method can predict several cleavage sites in pre-miRNA with different SVM scores; the highest score supposed to be the most probable cleavage site. Further, we suggest that users should consider top 3 predictions (based on scores), not only one top hit, as the only possible cleavage site. This is due to the fact that many isoforms of mature miRNAs are generated from same pre-miRNA sequence due to slight variation in the Drosha and Dicer cleavage site [44]. In this work, we have considered human pre-miRNAs. However, since most miRNAs, Dicer and its associated proteins are conserved across closely related organisms, we consider that our method could be also used for predicting Dicer cleavage site in even the related organisms like chimpanzee, rat or mice.

## Conclusions

From the present investigation, a method has been developed for the first time to predict Dicer cleavage site in the pre-miRNAs with 86.40% accuracy. Our method, PHDcleav, is available at http://www.imtech.res.in/raghava/phdcleav. Preliminary analysis indicates the lack of conserved sequence at the cleavage site since di- and tri-nucleotide features does not improve the performance than that of mono-nucleotide composition. Interestingly, when secondary structure features of cleavage sites were integrated using extended binary pattern, model accuracy drastically improved. This suggests the role of position specific nucleotides as well as structural characteristics in the recognition of Dicer cleavage site. Furthermore, we found better performance for cleavage site at the 5p arm than that of 3p arm. This improvement may be due to the presence of better discriminatory features at 5p arm compared to 3p arm and thus suggesting an urgent need to address them experimentally. The PHDcleav method has already been applied in various studies such as; polymorphic effect on Dicer cleave site [43], to find the size of mature miRNA [49], and in discovery of new miRNAs in the genome [50].

We believe that PHDcleav have the potential to be used in future investigations on genetic variations in miRNA loci and their effect on speed and accuracy of Dicer processing, and its impact on target gene silencing. Furthermore, studies have shown that use of Dicer specific siRNA can improve the RNAi silencing [65,66]. This tool will also be useful in the design and careful selection of shRNA/amiRNA for more potent gene silencing.

## Competing interests

The authors declare that they have no competing interests.

## Authors' contributions

FA and RK collected the data, implemented SVM for models development. FA wrote the manuscript and developed the PHDcleav server; RK edited the manuscript. GPSR designed and supervised the whole project, carried out data interpretation, and finalized the writing. All authors read and approved the final manuscript.

## Supplementary Material

Additional file 1 **This file contains supplementary information of methods, web server, figures and tables referred to in the text**. Click here for file

## Declarations

Publication of this work was funded by the CSIR under project Facility Creation (FAC-003) for Advanced Centre for Protein Informatics, Science and Technology.

This article has been published as part of *BMC Bioinformatics *Volume 14 Supplement 14, 2013: Proceedings of the Tenth Annual MCBIOS Conference. Discovery in a sea of data. The full contents of the supplement are available online at http://www.biomedcentral.com/bmcbioinformatics/supplements/14/S14.
