# Supplementary material for: PHDcleav: a SVM based method for predicting human Dicer cleavage sites using sequence and secondary structure of miRNA precursors
Source: BMC Bioinformatics. 2013 Oct 9;14(Suppl 14):S9. doi: 10.1186/1471-2105-14-S14-S9 (PMC3851333; doi:10.1186/1471-2105-14-S14-S9)
Supplement: Additional file 1 — This file contains supplementary information of methods, web server, figures and tables referred to in the text. [file 1471-2105-14-S14-S9-S1.pdf]

PHDcleav: A SVM based method for predicting human Dicer cleavage sites using sequence and secondary structure of miRNA precursors

Firoz Ahmed, Rakesh Kaundal and Gajendra PS Raghava

Additional file 1

Methods

Datasets

Training datasets contain 555 patterns of Dicer cleavage site (positive class) and 555 patterns of Dicer non-cleavage sites (negative class). We used non-redundant five-fold cross validation technique to evaluate the models. Many miRNA precursors are very similar to each other thus randomly dividing the pattern into 5 sets generates positive bias in the SVMs estimated performance. Therefore, in this study we placed all similar sequences, according to miFam.dat of miRBase into similar set.

Independent testing dataset contain 135 patters of Dicer cleavage and 135 patterns of Dicer non-cleavage.

Dicer cleavage sites

We considered the 3’ end of mature miRNA generated from 5’ arm and 5’ end of mature miRNA generated from 3’ arm as Dicer cleavage site. As the Dicer generally making 2 nt overhang at 3’ end, in case of miRNA generated from only 3’ arm we defined the Dicer cleavage site at 5’ arm with the help of 3’ arm. Similarly when the mature miRNA generated from only 5’ arm the Dicer cleavage site at 3’ arm is defined using 2 nt overhang in the structure.

Sequence pattern of Dicer cleavage site

We used the various length of Dicer cleavage pattern 8 nt, 10 nt, 12 nt and 14 nt to check the performance. Each pattern contains Dicer cleavage site at the center. Nucleotide

composition was taken as an input feature for SVM. In case of composition, vector size for SVM remains the same for different length of pattern like 4 dimensions for mononucleotide of 14 nt as well as 12 nt. While in case of binary pattern, vector size varied according to the length of Dicer pattern. Like 48 dimension for 12nt, and 56 dimensions for 14 nt.

Structure pattern of Dicer cleavage site

Here, in addition to the sequence pattern, we also included the information of their complementary base. In this case, a 14 nt pattern will contain 28 base (14 additional base from complementary strand) and thus represented by a vector of 112 dimension. We used two different methods for secondary structure: (1) structure from miRNA.str file of miRBase 13., and (2) generated the secondary structure of miRNA hairpin by using quikfold server (version 3.0 RNA rules) available at http://mfold.rna.albany.edu/?q=DINAMelt/Quickfold and structure having lowest free energy were taken.

Description of PHDcleav web server

Based on this study, the best model was used to develop the web server, PHDcleav (http://www.imtech.res.in/raghava/phdcleav/) for predicting Dicer cleavage site at 5' arm of human pre-miRNA. This is a user-friendly server developed on SUN server under Solaris environment using HTML, PERL and CGI-PERL.

Input: The input sequence for predicting Dicer cleavage site in pre-miRNA is a one-letter code nucleotide (A, C, G, U) in simple format. More than one sequence is separated by semicolon (;). The sequence can be pasted in the provided text area or can be uploaded through a file.

Options: The threshold is used to discriminate the dicer processing sites. The threshold should be chosen as 0.2, but user can vary the threshold score between -1.0 to 1.0.

Output: This algorithm extracts 14 nt long sliding structure pattern having cleavage site at the center and binary pattern generated. It checks the score of cleavage site from 17 to hairpin-loop on 5' arm of pre-miRNA. This is because lengths of miRNA vary from 17 nt to 27 nt (miRBase 13). If the score of the sequence is more than the threshold, the middle of the 14 nt is predicted as Dicer cleavage sites. The results may generate several cleavage sites with different SVM scores at a given site; highest score is considered as the most probable cleavage site. Each of the results display format firstly provides name of sequence, length of sequence, structure pattern having cleavage site at center followed by position of Dicer processing sites. One pre-miRNA sequence may generate different patterns and their corresponding SVM score. The secondary structure of each hairpin could be downloaded by clicking at the corresponding name.

(A) has-mir-200c

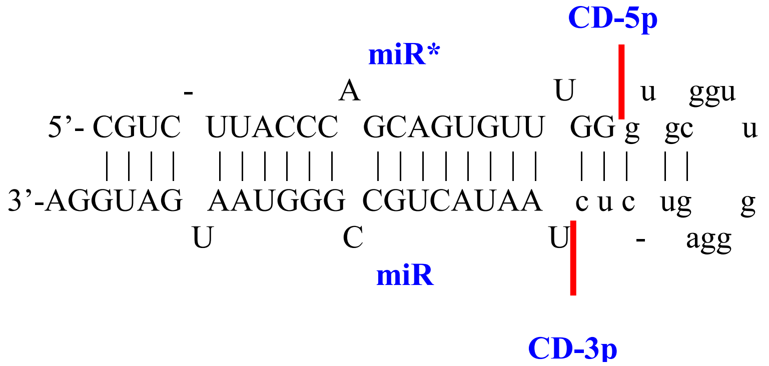

(B) Sequence of CP-5p: UGUUUGGgugcggu

+1 1:0.00 2:7.14 3:50.00 4:42.86

+1 1:0 2:0 3:0 4:1 5:0 6:0 7:1 8:0 …… 49:0 50:0 51:1 52:0 53:0 54:0 55:0 56:1

(C) Sequence of nCP-5p: C-UUACCCAGCAGU

-1 1:21.43 2:35.71 3:14.29 4:21.43

-1 1:0 2:1 3:0 4:0 5:0 6:0 7:0 8:0 ….. 49:0 50:0 51:1 52:0 53:0 54:0 55:0 56:1

(D) Structure of CP-5p:

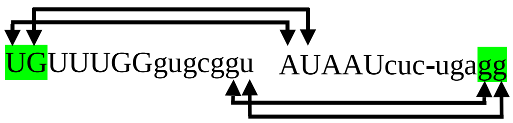

+1 1:14.29 2:10.71 3:35.71 4:35.71

+1 1:0 2:0 3:0 4:1 5:0 6:0 7:1 8:0 ….. 105:0 106:0 107:1 108:0 109:0 110:0 111:1 112:0

(E) Structure of nCP-5p: C-UUACCCAGCAGU GUAAUGGGCCGUCA

-1 1:21.43 2:28.57 3:25.00 4:21.43

-1 1:0 2:1 3:0 4:0 5:0 6:0 7:0 8:0…. 105:0 106:1 107:0 108:0 109:1 110:0 111:0 112:0

Figure S1: Schematic diagram of pre-miRNA, hsa-mir-200c, taken from miRNA.str of miRBase and patterns of Dicer cleavage site at 5' arm of hairpin. (A) miR* derived from 5' arm and miR derived from 3' arm of hairpin, bases presented in miR/miR* are represented in capital letter. CD-5p and CD-3p are cleavage site of Dicer at 5' and 3' arm respectively. (B) Sequence of CP-5p cleavage pattern of 14 nucleotides having cleavage

site CD-5p at centre. Following each cleavage pattern, feature of mononucleotide, and binary of the pattern used as input feature for SVM are given. (C) Sequence of nCP-5p non-cleavage pattern of 14 nucleotide derived from 5’ arm of pre-miRNA after omitting six nucleotide from CD-5p. (D) Structure of CP-5p cleavage pattern of 14 nucleotides having cleavage site CD-5p at centre and its partially complementary strand. Base of 5’ arm corresponding to 3’ arm are indicated with arrows. The pattern of 14+14 is used to generate binary pattern. (E) Structure of nCP-5p non-cleavage pattern of 14 nucleotide derived from 5’ arm and corresponding base of 3’ arm. Mononucleotide having 4, sequence binary pattern having 56, and structure binary pattern having 112 dimensional vector. +1 is the class for cleavage pattern and -1 is the class for non-cleavage pattern. Binary pattern is represented only for highlighted nucleotides.

(A) has-mir-200c

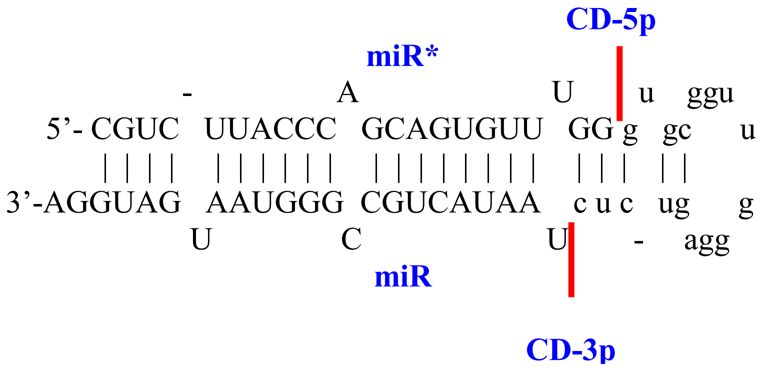

(B) Sequence of CP-3p: **UCAUAAUcuc-uga**

+1 1:28.57 2:21.43 3:7.14 4:35.71

+1 1:0 2:0 3:0 4:1 5:0 6:1 7:0 8:0 …… 49:0 50:0 51:1 52:0 53:1 54:0 55:0 56:0

(C) Sequence of nCP-3p: UAGUAAUGGGCCGU

-1 1:21.43 2:14.29 3:35.71 4:28.57

-1 1:0 2:0 3:0 4:1 5:1 6:0 7:0 8:0…… 49:0 50:0 51:1 52:0 53:0 54:0 55:0 56:1

(D) Structure of CP-3p: AGUGUUUGGgugcgUCAUAAUcuc-uga

+1 1:17.86 2:14.29 3:28.57 4:35.71

+1 1:1 2:0 3:0 4:0 5:0 6:0 7:1 8:0 ….. 105:0 106:0 107:1 108:0 109:1 110:0 111:0 112:0

(E) Structure of nCP-3p: GUC-UUACCCAGCAUAGUAAUGGGCCGU

-1 1:21.43 2:25.00 3:25.00 4:25.00

-1 1:0 2:0 3:1 4:0 5:0 6:0 7:0 8:1….. 105:0 106:0 107:1 108:0 109:0 110:0 111:0 112:1

Figure S2: Schematic diagram of pre-miRNA, hsa-mir-200c, taken from miRNA.str of miRBase and patterns of Dicer cleavage site at 3' arm of hairpin. (A) miR* derived from 5' arm and miR derived from 3 arm of hairpin, bases presented in miR/miR* are represented in capital letter. CD-5p and CD-3p are cleavage site of Dicer at 5' and 3' arm respectively. (B) Sequence of CP-3p cleavage pattern of 14 nucleotides having cleavage site CD-3p at centre. Following each cleavage pattern, feature of mononucleotide and binary of the pattern used as input feature for SVM are given. (C) Sequence of nCP-3p non-cleavage pattern of 14 nucleotide derived from 3' arm of pre-miRNA after omitting

six nucleotide from CD-3p. (D) Structure of CP-3p cleavage pattern of 14 nucleotides having cleavage site CD-3p at centre and its partially complementary strand. The pattern of 14+14 is used to generate binary pattern. (E) Structure of nCP-3p non-cleavage pattern of 14 nucleotide derived from 3’ arm and corresponding base of 5’ arm. Mononucleotide having 4, sequence binary pattern having 56, and structure binary pattern having 112 dimensional vector. +1 is the class for cleavage pattern and -1 is the class for non-cleavage pattern. Binary pattern is represented only for highlighted nucleotides.

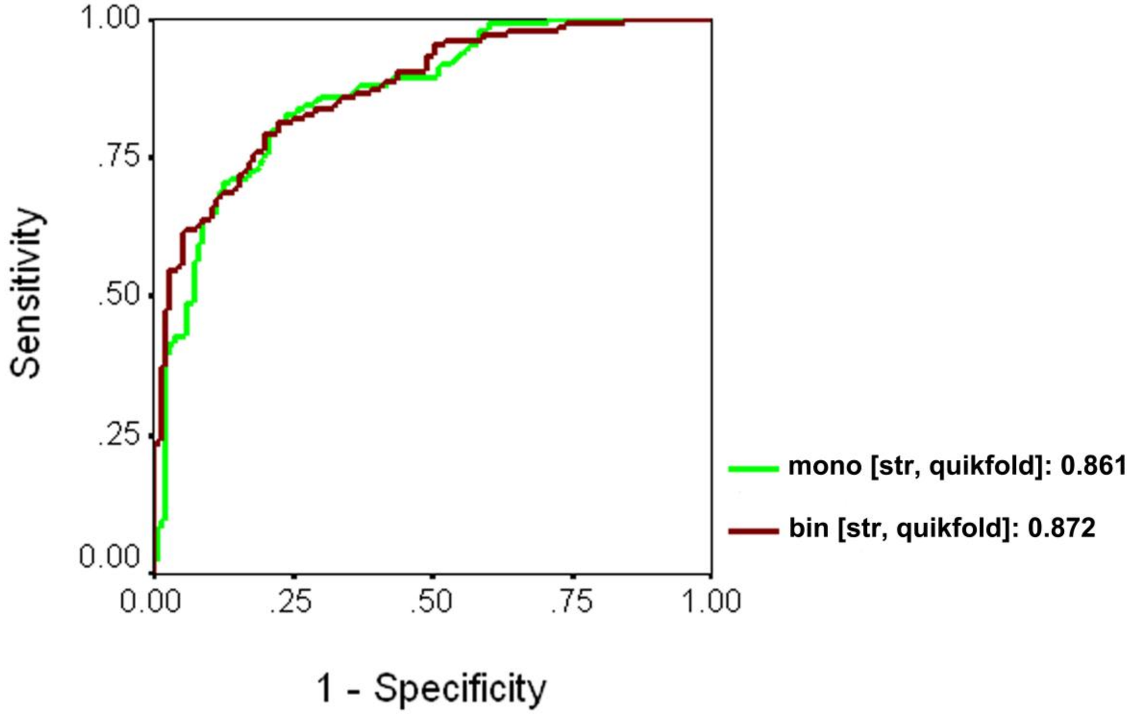

Figure S3: Performance of two best SVM models for Dicer cleavage site at 5p arm (CD-5p) on an independent dataset containing 135 pre-miRNA. The value indicates AUC for the corresponding model.

Table S1: Performance of SVM-based model for Dicer cleavage site at 5p arm **(structure of CP-5p)** developed using **binary pattern** feature. SVM^{light} parameters: g:0.01, c:8, j:2.

Th	TP	TN	FP	FN	Sn	Sp	Ac	Mc
-1	541	177	378	14	97.48	31.89	64.68	0.39
-0.9	538	195	360	17	96.94	35.14	66.04	0.41
-0.8	536	232	323	19	96.58	41.80	69.19	0.46
-0.7	531	266	289	24	95.68	47.93	71.80	0.50
-0.6	524	293	262	31	94.41	52.79	73.60	0.52
-0.5	519	330	225	36	93.51	59.46	76.49	0.56
-0.4	515	351	204	40	92.79	63.24	78.02	0.59
-0.3	510	384	171	45	91.89	69.19	80.54	0.63
-0.2	501	406	149	54	90.27	73.15	81.71	0.64
-0.1	495	430	125	60	89.19	77.48	83.33	0.67
0	487	449	106	68	87.75	80.90	84.32	0.69
0.1	480	470	85	75	86.49	84.68	85.59	0.71
**0.2**	**473**	**484**	**71**	**82**	**85.23**	**87.21**	**86.22**	**0.72**
0.3	457	496	59	98	82.34	89.37	85.86	0.72
0.4	443	506	49	112	79.82	91.17	85.50	0.71
0.5	419	516	39	136	75.50	92.97	84.23	0.70
0.6	400	523	32	155	72.07	94.23	83.15	0.68
0.7	377	528	27	178	67.93	95.14	81.53	0.66
0.8	358	536	19	197	64.50	96.58	80.54	0.64
0.9	343	538	17	212	61.80	96.94	79.37	0.63
1	328	542	13	227	59.10	97.66	78.38	0.62

Th: Threshold, Sn: sensitivity, Sp: specificity, Ac: accuracy, Mc: Matthews correlation coefficient.

Table S2: Performance of Dicer cleavage site at 5p arm on independent dataset of 135 pre-miRNA sequences on SVM model (Table S1) trained on 555 pre-miRNA sequences using binary pattern feature.

Th	TP	TN	FP	FN	Sn	Sp	Ac	Mc
-1	134	32	103	1	99.26	23.70	61.48	0.35
-0.9	133	37	98	2	98.52	27.41	62.96	0.37
-0.8	132	46	89	3	97.78	34.07	65.93	0.41
-0.7	130	56	79	5	96.30	41.48	68.89	0.45
-0.6	129	65	70	6	95.56	48.15	71.85	0.50
-0.5	123	69	66	12	91.11	51.11	71.11	0.46
-0.4	122	76	59	13	90.37	56.30	73.33	0.50
-0.3	119	80	55	16	88.15	59.26	73.70	0.50
-0.2	116	86	49	19	85.93	63.70	74.81	0.51
-0.1	114	91	44	21	84.44	67.41	75.93	0.53
0	112	96	39	23	82.96	71.11	77.04	0.54
0.1	110	105	30	25	81.48	77.78	79.63	0.59
**0.2**	**103**	**108**	**27**	**32**	**76.30**	**80.00**	**78.15**	**0.56**
0.3	98	112	23	37	72.59	82.96	77.78	0.56
0.4	94	115	20	41	69.63	85.19	77.41	0.55
0.5	93	118	17	42	68.89	87.41	78.15	0.57
0.6	90	120	15	45	66.67	88.89	77.78	0.57
0.7	85	124	11	50	62.96	91.85	77.41	0.57
0.8	84	127	8	51	62.22	94.07	78.15	0.59
0.9	78	128	7	57	57.78	94.81	76.30	0.57
1	72	131	4	63	53.33	97.04	75.19	0.56

Table S3: Performance of SVM-based model ***(Model 1)*** for Dicer cleavage site at 5p arm **(structure of CP-5p)** developed using **extended binary pattern** feature. SVM^{light} parameters: g:0.01, c:8, j:1.

Th	TP	TN	FP	FN	Sn	Sp	Ac	sd(Ac)	Mc	sd(Mc)
-1	531	260	295	24	95.68	46.85	71.26	3.03	0.49	0.047
-0.9	525	292	263	30	94.59	52.61	73.60	2.81	0.52	0.04
-0.8	521	333	222	34	93.87	60.00	76.94	2.52	0.57	0.043
-0.7	517	351	204	38	93.15	63.24	78.20	1.95	0.59	0.036
-0.6	513	376	179	42	92.43	67.75	80.09	1.75	0.62	0.034
-0.5	507	393	162	48	91.35	70.81	81.08	2.49	0.64	0.052
-0.4	496	422	133	59	89.37	76.04	82.70	2.58	0.66	0.055
-0.3	488	441	114	67	87.93	79.46	83.69	2.03	0.68	0.042
-0.2	482	460	95	73	86.85	82.88	84.86	2.18	0.70	0.043
-0.1	476	476	79	79	85.77	85.77	85.77	2.25	0.72	0.042
**0**	**468**	**491**	**64**	**87**	**84.32**	**88.47**	**86.40**	**1.29**	**0.73**	**0.027**
0.1	457	499	56	98	82.34	89.91	86.13	1.33	0.72	0.027
0.2	445	508	47	110	80.18	91.53	85.86	1.98	0.72	0.036
0.3	430	516	39	125	77.48	92.97	85.23	3.57	0.71	0.062
0.4	415	521	34	140	74.77	93.87	84.32	3.84	0.70	0.063
0.5	404	523	32	151	72.79	94.23	83.51	4.26	0.69	0.069
0.6	389	529	26	166	70.09	95.32	82.70	4.15	0.68	0.066
0.7	372	535	20	183	67.03	96.40	81.71	3.8	0.66	0.059
0.8	350	538	17	205	63.06	96.94	80.00	4.57	0.64	0.073
0.9	331	542	13	224	59.64	97.66	78.65	4.53	0.62	0.07
1	316	547	8	239	56.94	98.56	77.75	4.48	0.61	0.064

Th: Threshold, Sn: sensitivity, Sp: specificity, Ac: accuracy, Mc: Matthews correlation coefficient, sd: standard deviation

Table S4: Performance of Dicer cleavage site at 5p arm on independent dataset of 135 pre-miRNA sequences on SVM model (Table S3) trained on 555 pre-miRNA sequences using extended binary pattern feature ***(Model 1)***.

Th	TP	TN	FP	FN	Sn	Sp	Ac	Mc
-1	131	54	81	4	97.04	40.00	68.52	0.45
-0.9	129	65	70	6	95.56	48.15	71.85	0.50
-0.8	124	71	64	11	91.85	52.59	72.22	0.48
-0.7	123	77	58	12	91.11	57.04	74.07	0.51
-0.6	120	82	53	15	88.89	60.74	74.81	0.52
-0.5	117	89	46	18	86.67	65.93	76.30	0.54
-0.4	116	98	37	19	85.93	72.59	79.26	0.59
-0.3	114	101	34	21	84.44	74.81	79.63	0.60
-0.2	112	105	30	23	82.96	77.78	80.37	0.61
-0.1	110	108	27	25	81.48	80.00	80.74	0.61
**0**	**110**	**111**	**24**	**25**	**81.48**	**82.22**	**81.85**	**0.64**
0.1	103	115	20	32	76.30	85.19	80.74	0.62
0.2	99	118	17	36	73.33	87.41	80.37	0.61
0.3	96	121	14	39	71.11	89.63	80.37	0.62
0.4	91	123	12	44	67.41	91.11	79.26	0.60
0.5	89	126	9	46	65.93	93.33	79.63	0.62
0.6	82	128	7	53	60.74	94.81	77.78	0.59
0.7	77	131	4	58	57.04	97.04	77.04	0.59
0.8	74	132	3	61	54.81	97.78	76.30	0.58
0.9	71	132	3	64	52.59	97.78	75.19	0.56
1	68	132	3	67	50.37	97.78	74.07	0.55

Table S5: Performance of SVM-based model ***(Model 2)*** for Dicer cleavage site at 5p arm **(structure of CP-5p)** developed using **extended binary pattern** feature. Training dataset contains of 555 positive patterns and 18662 negative patterns. SVM^{light} parameters: g:0.001, c:2, j:10.

Th	TP	TN	FP	FN	Sn	Sp	Ac	Mc
-1	449	13825	4837	106	80.90	74.08	74.28	0.21
-0.9	439	14406	4256	116	79.10	77.19	77.25	0.22
-0.8	417	14929	3733	138	75.14	80.00	79.86	0.22
-0.7	408	15380	3282	147	73.51	82.41	82.16	0.24
-0.6	388	15796	2866	167	69.91	84.64	84.22	0.24
-0.5	360	16162	2500	195	64.86	86.60	85.98	0.24
**-0.4**	**346**	**16496**	**2166**	**209**	**62.34**	**88.39**	**87.64**	**0.25**
-0.3	326	16796	1866	229	58.74	90.00	89.10	0.26
-0.2	301	17070	1592	254	54.23	91.47	90.39	0.26
-0.1	285	17285	1377	270	51.35	92.62	91.43	0.26
0	262	17518	1144	293	47.21	93.87	92.52	0.26
0.1	233	17686	976	322	41.98	94.77	93.25	0.25
0.2	206	17874	788	349	37.12	95.78	94.08	0.25
0.3	183	18021	641	372	32.97	96.57	94.73	0.24
0.4	164	18141	521	391	29.55	97.21	95.25	0.24
0.5	145	18249	413	410	26.13	97.79	95.72	0.24
0.6	132	18320	342	423	23.78	98.17	96.02	0.24
0.7	113	18385	277	442	20.36	98.52	96.26	0.22
0.8	93	18454	208	462	16.76	98.89	96.51	0.21
0.9	73	18512	150	482	13.15	99.20	96.71	0.19
1	60	18556	106	495	10.81	99.43	96.87	0.19

Table S6: Performance of SVM-based model ***(Model 2^{balanced})*** for Dicer cleavage site at 5p arm **(structure of CP-5p)** developed using **extended binary pattern** feature. Training dataset contains of 555 positive patterns and 555 negative patterns randomly selected from 18662. SVM^{light} parameters: g:0.001, c:1, j:1.

Th	TP	TN	FP	FN	Sn	Sp	Ac	Mc
-1	549	53	502	6	98.92	9.55	54.23	0.19
-0.9	542	82	473	13	97.66	14.77	56.22	0.22
-0.8	533	138	417	22	96.04	24.86	60.45	0.3
-0.7	526	170	385	29	94.77	30.63	62.70	0.33
-0.6	518	221	334	37	93.33	39.82	66.58	0.39
-0.5	503	258	297	52	90.63	46.49	68.56	0.41
-0.4	491	306	249	64	88.47	55.14	71.80	0.46
-0.3	475	343	212	80	85.59	61.80	73.69	0.49
-0.2	454	378	177	101	81.8	68.11	74.95	0.50
**-0.1**	**441**	**406**	**149**	**114**	**79.46**	**73.15**	**76.31**	**0.53**
0	415	427	128	140	74.77	76.94	75.86	0.52
0.1	382	448	107	173	68.83	80.72	74.77	0.50
0.2	338	464	91	217	60.90	83.60	72.25	0.46
0.3	307	487	68	248	55.32	87.75	71.53	0.46
0.4	280	510	45	275	50.45	91.89	71.17	0.47
0.5	230	519	36	325	41.44	93.51	67.48	0.41
0.6	191	528	27	364	34.41	95.14	64.77	0.37
0.7	146	533	22	409	26.31	96.04	61.17	0.31
0.8	107	541	14	448	19.28	97.48	58.38	0.27
0.9	79	545	10	476	14.23	98.20	56.22	0.23
1	54	550	5	501	9.73	99.10	54.41	0.20

Table S7: Pre-miRNA taken from release (version 14) but not present in the last release (version 13) of miRBase and named as exclusive miRBase 14. Performance of SVM models was assessed on these 30 sequences not used in training datasets.

Sn	Pre-miRNA
1	hsa-mir-147
2	hsa-mir-190b
3	hsa-mir-208a
4	hsa-mir-211
5	hsa-mir-220a
6	hsa-mir-220b
7	hsa-mir-298
8	hsa-mir-300
9	hsa-mir-325
10	hsa-mir-346
11	hsa-mir-384
12	hsa-mir-412
13	hsa-mir-449c
14	hsa-mir-513b
15	hsa-mir-513c
16	hsa-mir-548q
17	hsa-mir-670
18	hsa-mir-711
19	hsa-mir-718
20	hsa-mir-759
21	hsa-mir-761
22	hsa-mir-762
23	hsa-mir-764
24	hsa-mir-2114
25	hsa-mir-2115
26	hsa-mir-2116
27	hsa-mir-2117
28	hsa-mir-2276
29	hsa-mir-2277
30	hsa-mir-2278

Table S8: Performance of Dicer cleavage site at 5p arm on independent dataset of 30 pre-miRNA sequences **(exclusive miRBase 14)** on ***Model 1*** (Table S3) trained on 555 positive and 555 negative patterns using extended binary pattern feature. Top three predicted cleavages site were compared with the actual cleavage site and calculated the PSE valus =(Act Pos- Pred Pos); PSE which is most close to actual cleavage site among three PSE is denoted as close PSE. PSE: position shift error, Act: actual, Pred: predicted.

**Sn**	**Act Pos**	**1st Top score**		**2nd Top score**		**3rd Top Score**		**PSE (Act Pos- Pred Pos)**			**Close PSE**
		Pos	score	Pos	score	Pos	score	**1st Top score**	**2nd Top score**	**3rd Top Score**	
1	28	30	2.112	28	1.362	23	0.937	2	0	-5	0
2	31	33	1.780	34	1.754	32	1.647	2	3	1	1
3	31	32	1.621	30	1.213	31	1.032	1	-1	0	0
4	47	51	1.479	49	1.403	52	1.135	4	2	5	2
5	43	42	1.365	47	1.127	46	0.986	-1	4	3	1
6	31	31	1.154	39	0.626	36	0.378	0	8	5	0
7	34	38	1.503	36	0.808	35	0.597	4	2	1	1
8	29	36	3.375	35	2.221	32	1.597	7	6	3	3
9	38	38	1.058	36	0.544	37	0.408	0	-2	-1	0
10	42	44	3.416	43	2.255	42	1.623	2	1	0	0
11	32	39	1.697	36	1.303	38	1.224	7	4	6	4
12	40	40	1.128	39	0.855	31	0.548	0	-1	-9	0
13	41	42	2.235	43	1.713	41	1.277	1	2	0	0
14	35	35	2.562	37	2.452	36	1.926	0	2	1	0
15	35	35	2.509	37	2.452	36	2.063	0	2	1	0
16	29	49	2.932	48	2.469	47	2.020	20	19	18	18
17	39	46	2.668	45	2.034	44	1.899	7	6	5	5
18	32	32	2.159	33	1.063	31	0.898	0	1	-1	0
19	32	25	0.069	31	-0.089	32	-0.184	-7	-1	0	0
20	45	43	2.112	44	1.507	40	1.109	-2	-1	-5	1
21	28	25	1.581	24	1.211	28	0.907	-3	-4	0	0
22	35	36	0.312	39	0.181	35	-0.191	1	4	0	0
23	32	40	1.518	37	1.362	39	0.993	8	5	7	5
24	34	35	2.170	34	1.922	33	1.628	1	0	-1	0
25	42	42	2.369	41	2.123	40	1.689	0	-1	-2	0
26	34	36	1.385	33	1.196	35	0.729	2	-1	1	1
27	35	35	2.099	34	1.585	33	0.435	0	-1	-2	0
28	37	40	2.523	41	1.732	39	1.507	3	4	2	2
29	40	41	3.322	40	1.853	39	1.185	1	0	-1	0
30	37	44	1.585	43	0.627	40	0.514	7	6	3	3
Average PSE (Position Shift Error)								3.1	3.13	2.9	1.56
Average PSE (Excluding SN 18)								2.52	2.59	2.45	1
# with actual position (PSE 0)								8	3	6	17
# with 1nt deviation (PSE 1)								6	9	9	5
# with 2nt deviation (PSE 2)								5	6	3	2
# with 3nt deviation (PSE 3)								2	1	3	2
# upto 3nt deviation/total sequence								21/30	19/30	21/30	26/30

Table S9: Performance of Dicer cleavage site at 5p arm on independent dataset of 30 pre-miRNA sequences **(exclusive miRBase 14)** on ***Model 2*** (Table S5) trained on 555 positive and 18662 negative patterns using extended binary pattern feature. Top three predicted cleavages site were compared with the actual cleavage site and calculated the PSE valus =(Act Pos- Pred Pos); PSE which is most close to actual cleavage site among three PSE is denoted as close PSE. PSE: position shift error, Act: actual, Pred: predicted.

**Sn**	**Act Pos**	**1st Top score**		**2nd Top score**		**3rd Top Score**		**PSE (Act Pos- Pred Pos)**			**Close PSE**
		Pos	score	Pos	score	Pos	Score	**1st Top score**	**2nd Top score**	**3rd Top Score**	
1	28	30	0.264	32	-0.636	20	-0.700	2	4	-8	2
2	31	33	0.517	34	0.366	32	0.284	2	3	1	1
3	31	31	0.596	30	0.132	32	-0.074	0	-1	1	0
4	47	46	0.126	47	-0.043	48	-0.338	-1	0	1	0
5	43	47	-0.047	46	-0.277	41	-0.557	4	3	-2	2
6	31	36	-0.688	31	-0.741	35	-0.760	5	0	4	0
7	34	38	0.375	35	-0.452	37	-0.472	4	1	3	1
8	29	36	2.164	35	0.844	27	-0.229	7	6	-2	2
9	38	38	-0.291	34	-0.661	35	-0.673	0	-4	-3	0
10	42	44	1.558	43	0.834	40	0.226	2	1	-2	1
11	32	39	1.335	38	-0.172	28	-0.250	7	6	-4	4
12	40	40	-0.100	42	-0.148	39	-0.336	0	2	-1	0
13	41	42	0.555	43	0.420	44	0.090	1	2	3	1
14	35	35	1.328	36	1.066	37	0.677	0	1	2	0
15	35	36	1.161	35	0.919	37	0.677	1	0	2	0
16	29	48	1.244	49	1.094	47	1.094	19	20	18	19
17	39	43	0.817	42	0.432	46	0.345	4	3	7	3
18	32	32	-0.208	31	-0.621	33	-0.629	0	-1	1	0
19	32	29	-1.084	24	-1.285	30	-1.302	-3	-8	-2	2
20	45	43	0.836	44	-0.111	40	-0.158	-2	-1	-5	1
21	28	24	-0.192	25	-0.251	26	-0.438	-4	-3	-2	2
22	35	36	-0.520	35	-0.885	39	-0.981	1	0	4	0
23	32	40	0.547	39	-0.117	38	-0.297	8	7	6	6
24	34	35	0.923	34	0.434	33	-0.079	1	0	-1	0
25	42	41	1.153	42	0.870	40	0.415	-1	0	-2	0
26	34	36	0.526	33	-0.387	34	-0.644	2	-1	0	0
27	35	35	1.235	34	0.012	33	-0.501	0	-1	-2	0
28	37	39	-0.266	38	-0.337	40	-0.408	2	1	3	1
29	40	41	1.076	40	0.203	37	-0.085	1	0	-3	0
30	37	44	-0.080	43	-0.481	40	-0.575	7	6	3	3
Average PSE								3.03	2.87	3.27	1.7
Average PSE (Excluding SN 18)								2.48	2.28	2.76	1.1
# with actual position (PSE 0)								6	7	1	14
# with 1nt deviation (PSE 1)								7	9	6	6
# with 2nt deviation (PSE 2)								6	2	9	5
# with 3nt deviation (PSE 3)								1	4	6	2
# upto 3nt deviation/total sequence								20/30	22/30	22/30	27/30
